# PKM2 Facilitates Classical Swine Fever Virus Replication by Enhancing NS5B Polymerase Function

**DOI:** 10.3390/v17050648

**Published:** 2025-04-29

**Authors:** Mengzhao Song, Shanchuan Liu, Yan Luo, Tiantian Ji, Yanming Zhang, Wen Deng

**Affiliations:** College of Veterinary Medicine, Northwest A&F University, Yangling 712100, China; songmengzhao@nwafu.edu.cn (M.S.); liushanchuan@nwafu.edu.cn (S.L.); luoyan123@nwafu.edu.cn (Y.L.); jtt@nwafu.edu.cn (T.J.)

**Keywords:** classical swine fever virus, pyruvate kinase M2, viral infection, NS5B, RdRp activity

## Abstract

Host metabolic reprogramming is a critical strategy employed by many viruses to support their replication, and the key metabolic enzyme plays important roles in virus infection. This study investigates the role of pyruvate kinase M2 (PKM2), a glycolytic enzyme with non-canonical functions, in the replication of classical swine fever virus (CSFV). Using PK-15 cells and piglet models, we demonstrate that CSFV infection upregulates PKM2 expression both in vitro and in vivo, creating a proviral environment. knockdown of PKM2 by siRNA reduced CSFV proliferation, while PKM2 overexpression significantly increased virus propagation, which was evaluated by viral protein synthesis, genome replication, and progeny virion production. A direct interaction between PKM2 and CSFV NS5B protein was identified by co-immunoprecipitation and GST-pulldown assays, and PKM2 affected NS5B polymerase activity in a dual-luciferase reporter assay, with PKM2 depletion reducing RdRp function by 50%. Temporal analysis of the first viral replication cycle confirmed PKM2-dependent enhancement of CSFV RNA synthesis. These findings establish PKM2 as a proviral host factor that directly binds NS5B to potentiate RdRp activity, thereby bridging metabolic adaptation and viral genome replication. This study provides new evidence of a glycolytic enzyme physically interacting and enhancing viral polymerase function, offering new information about CSFV–host interaction.

## 1. Introduction

Classical swine fever virus (CSFV) is the pathogen causing classical swine fever, an epizootic disease of pigs that impairs the pig industry worldwide and is listed in the Terrestrial Animal Health Code of the WOAH. Belonging to the Pestivirus suis species within the Pestivirus genus of the *Flaviviridae* family [1], the CSFV genome is a single-stranded positive-sense RNA with one large open reading frame (ORF) flanked by 5’ and 3’ untranslated regions (UTR) [2]. This genome encodes a 3898 amino acid precursor polypeptide that is cleaved into four structural proteins (C, E^rns^, E1, and E2) and eight non-structural proteins (N^pro^, p7, NS2, NS3, NS4A, NS4B, NS5A, and NS5B) [3]. NS5B is the RNA-dependent RNA polymerase (RdRp) of CSFV, regulating viral genome replication [4]. When the CSFV genome is transcribed into negative-strand RNA, NS5B binds to the negative-strand RNA to produce positive-strand RNA copies for the progeny virus [5], and other RNA templates can also be used by NS5B for RNA synthesis when tested in vitro [6].

Pyruvate kinase is the enzyme that catalyzes the final step of glycolysis by transferring a phosphate group from phosphoenolpyruvate (PEP) to ADP, generating pyruvate and ATP. The vertebrate pyruvate kinase generally has two types: the LR type, which is encoded by the PKLR gene and mainly expressed in liver and erythrocytes, and the M type, which is encoded by the PKM (pyruvate kinase muscle) gene and is ubiquitously expressed in proliferative cells with a high amount in muscle [7]. The human M type pyruvate kinase gene transcribes into two splicing isoforms, PKM1 and PKM2, which is also the case in pigs. While PKM1 constitutively maintains tetrameric structures for sustained glycolytic activity, PKM2 dynamically shifts between active tetramers and less-active dimers/monomers, acting as a metabolic sensor to balance energy production with other biosynthetic precursor production during multiple physiological and pathological processes, such as in tumorigenesis and apoptosis [8,9]. Therefore, the function and regulation mechanism of PKM2 in cancer initiation and proliferation has been intensively studied in recent years, making it a significant target in oncological studies. 

As a key enzyme for ATP production, it is not surprising that PKM2 has been reported to participate in virus infection since ATP is the molecule responsible for producing energy for most biological processes. On top of that, pyruvate kinase has been shown to interact with the RdRp of hepatitis virus C (HCV), influenza virus, and tomato bushy virus (TBSV) [10,11,12], and PKM2 has been shown to participate in the proliferation of viruses from different families. In CSFV infection, cellular glycolysis is upregulated [13], and the PKM2 has been shown to induce mitophagy and promote CSFV proliferation [14], and reduction of CSFV propagation resulting from PKM2 knockdown could be partially rescued by the addition of pyruvate, showing an enzymatic activity-dependent role of PKM2 in CSFV infection. 

The full role of PKM2 in CSFV infection remains elusive. In the current study, we explored the function of PKM2 on CSFV infection and its underlying mechanism and revealed direct regulation of the viral NS5B protein by PKM2, offering new information on pyruvate kinase and virus interactions.

## 2. Materials and Methods

### 2.1. Cells, Virus and Antibodies

Porcine kidney (PK-15) and the swine umbilical vein endothelial (SUVECs) cell lines were cultured in Medium 199 (Gibco, Shanghai, China) supplemented with 10% fetal bovine serum (FBS; Gibco, Shanghai, China) and penicillin-streptomycin solution (Sigma-Aldrich, Shanghai, China). The CSFV Shimen strain was obtained from the China Institute of Veterinary Drug Control (Beijing, China) and was propagated in PK-15 cells. Anti-β-actin rabbit antibody, anti-PKM2 rabbit monoclonal antibody, anti-GFP antibody, and anti-GST antibody were purchased from Abcam (Shanghai, China). Anti-E2 antibody was produced in mouse (Ab-mart, Shanghai, China). HRP-labeled goat anti-mouse and anti-rabbit IgG were obtained from Beyotime Biotechnology (Shanghai, China). Anti-Flag antibody was purchased from Proteintech (Wuhan, China). FITC-conjugated anti-mouse antibody was obtained from Sigma-Aldrich (Shanghai, China).

### 2.2. Plasmid Construction

The total RNA of processed cells was extracted using RNAiso Plus (Takara Bio, Beijing, China) and reverse transcribed into complementary DNA (cDNA) using an RT PCR Kit (Accurate Biotechnology, Hunan, China). Relative RNA expression was estimated using the TB Green™ Premix Ex Taq™ II (Takara Bio, Beijing, China) and evaluated using the Applied Biosystems 7500 Real-Time PCR system (Thermo Fisher Scientific, Waltham, MA, USA). PKM2, CSFV NS5B, and CSFV C encoding sequences were amplified by RT-PCR and separately inserted into pcDNA3.1-Flag and pcDNA3.1-GFP vectors to generate plasmids pcDNA3.1-PKM2-Flag, pcDNA3.1-NS5B-Flag, pcDNA3.1-C-Flag, pcDNA3.1-PKM2-GFP, pcDNA3.1-NS5B-GFP, and pcDNA3.1-C-GFP. Then, NS5B and C encoding sequences were inserted into pcDNA3.1-Red to create plasmids pcDNA3.1-NS5B-Red and pcDNA3.1-C-Red. PKM2, NS5B, and C encoding sequences were inserted into pGEX-6p-1 to produce fusion proteins pGEX-GST-PKM2, pGEX-GST-NS5B, and pGEX-GST-C. All primer sequences used in plasmid construction are shown in Table S1. Plasmid pCMV-RIG-I-3×Myc, IFN-β-pGL3, and pGL4.74[hRluc-TK] were purchased from Miaolin Biotechnology (Wuhan, China).

### 2.3. siRNA Transfection

siRNAs were synthesized by GenePharma (Shanghai, China), and sequences will be made available upon request. Cells were seeded in 12-well plates and transfected with relevant siRNA based on the manufacturers’ instructions. At 24 h after transfection, the efficacy of siPKM2 was detected by western blot and RT-qPCR [15], and the most effective one was selected for subsequent experiments.

### 2.4. Cell Viability Assay

The viability of transfected cells was measured using a Cell Counting Kit-8 (CCK-8) (Dojindo, Kumamoto, Japan) according to the manufacturer’s instructions. Briefly, PK-15 cells seeded in 96-well plates were transfected with relevant plasmid and siRNA and cultured for 72 h. Then, 10 μL of the cell viability reagent was added to each well, followed by incubation for 2 h at 37 °C. The absorbance values were recorded at 450 nm using a SpectraMax M5 Microplate Reader (Molecular Devices, San Francisco, CA, USA).

### 2.5. Transient Transfection of Plasmid

PK-15 cells were seeded in 6-well and 12-well plates until the confluency for adherent cells reached 70%. Plasmids were added into Opti-MEMTM (Thermo Fisher Scientific, Shanghai, China), followed by mixing with TurboFectTM Transfection Reagent (Thermo Fisher Scientific, Shanghai, China). After incubation for 20 min at room temperature, the mixture was added to each well. Transient transgene expression was obvious after 12 h post transfection.

### 2.6. Virus Titration by Indirect Immunofluorescence Assay (IFA)

PK-15 cells seeded in 96-well plates were infected with CSFV (MOI = 1). After 72 h, cells were fixed with 4% paraformaldehyde for 20 min, permeabilized by 0.5% Triton X-100 for 10 min, and treated with 3% BSA for 2 h, followed by overnight incubation with mouse anti-E2 antibody (Ab-mart, Shanghai, China) at 4 °C. Between each step, cells were washed three times with PBS. Subsequently, cells were incubated with FITC-conjugated anti-mouse antibody at 25 °C for 1 h. After washing, the fluorescence of positive wells was observed under a fluorescence inversion microscope (Nikon, Tokyo, Japan). The Reed and Muench method was used to determine the viral titers (TCID_50_) mL^−1^.

### 2.7. Western Blot

Protein samples were separated by 10% SDS-PAGE and transferred to polyvinylidene difluoride (PVDF) membranes (Merck Millipore, Shanghai, China), then blocked with 5% milk. Thereafter, membranes were incubated with primary antibodies at 4 °C overnight. After three washes with TBST, membranes were incubated with appropriate horseradish peroxidase (HRP)-conjugated secondary antibody at room temperature for 2 h. Protein expression was evaluated using an enhanced chemiluminescence (ECL) analysis system.

### 2.8. Confocal Microscopy

The PK-15 cells were seeded onto glass coverslips in 35 mm cell culture dishes overnight. Then, PK-15 cells were transfected with pcDNA3.1-NS5B-Red, pcDNA3.1-C-Red, or pcDNA3.1-Red, respectively. For virus infection, cells were infected with CSFV (MOI = 1) at 24 h post transfection. At 48 h post transfection, all cells, either with CSFV infection or not, were washed with cold PBS three times and fixed with 4% paraformaldehyde for 20 min. After washing with PBS, cells were permeabilized with 0.1% Triton X-100 for 10 min and blocked with 2% bovine serum albumin (BSA) for 2 h. Then, cells were incubated with mouse anti-PKM2 antibody (Abcam, Shanghai, China) at 4 °C for 12 h. After washing with PBS, cells were incubated with (FITC)-conjugated goat anti-mouse IgG at room temperature for 1 h. Subsequently, the cells were incubated with DAPI for 10 min at room temperature and washed with cold PBS. The fluorescence was observed under a laser scanning confocal microscope (LSM510 META, Carl Zeiss AG, Jena, Germany). DAPI was excited with a 405 nm laser, and FITC and DsRed were excited with 488 nm and 561 nm lasers, respectively. Emission fluorescence was filtered by bandpass filters, and then the signals were detected with a 63× oil objective and recorded by PMTs; digital images were formatted with a frame size of 1000 × 1000 pixels. Images were then processed with ImageJ software version 3.2 SP2.

### 2.9. Infection of Animals

This experiment used 6 Specific Pathogen-Free (SPF) pigs (30 days, 10–15 kg) from Chongqing Academy of Animal Sciences. The animal procedures were supervised by the Animal Ethical and Welfare Committee, Northwest A&F University (approval No. 2021042). Piglets were housed indoors and provided with water and food regularly. All of these pigs were confirmed to be negative for CSFV by using RT-qPCR and antibody (Ab) test kits (IDEXX). Then, 3 pigs were injected intramuscularly with 1 mL CSFV Shimen strain collected from infected SUVECs (10^5^ TCID_50_/mL); another 3 piglets were used as the negative control. After 21 days, all the infected piglets exhibited typical symptoms of CSF, such as the purulent secretion on conjunctiva, hematochezia, and hemorrhagic spots on the skin. In addition, the infected piglets could not eat food nor drink anymore. Therefore, all of these pigs were euthanized, and the tissues were collected for subsequent experiments. 

### 2.10. Immunohistochemistry (IHC)

Fresh tissue was fixed in paraformaldehyde (4%) for 24 h, followed by being soaked in ethanol with different concentrations. Then, the tissue was soaked in paraffin and trimmed by a paraffin slicer. The tissue slice was flattened in warm water at 40 °C and baked in an oven at 60 °C. The dried paraffin slices were removed and stored at room temperature. For IHC, the paraffin slices were sequentially incubated with xylene, anhydrous ethanol, and alcohol. After washing in distilled water, the tissue slices were placed in EDTA antigen retrieval buffer for antigen retrieval in a microwave oven. Then, samples were blocked by endogenous peroxide blocking solution and covered with 3% BSA. After that, the tissue was incubated with antibody in a wet box at 4 °C overnight, followed by wash with PBS. Finally, a freshly prepared DAB chromogenic solution was added, and nuclei were stained with hematoxylin. After incubation with alcohol, anhydrous ethanol, and xylene, the slices were used for microscopic examination.

### 2.11. Co-Immunoprecipitation (Co-IP) Assays

PK-15 cells cultured in 6-well plates were co-transfected with the indicated plasmids. After 48 h, transfected cells were lysed with RIPA lysis buffer (Beyotime Biotechnology, Shanghai, China) at 4 °C for 30 min, then centrifuged at 12,000× *g*, 4 °C, for 30 min to collect the supernatant. Anti-Flag magnetic beads (Bimake, Shanghai, China) were resuspended with TBS two times. The cell lysate was incubated with magnetic beads at 4 °C overnight. A magnet shelf was used to recollect magnet beads and discard supernatants. After washing with PBST, these beads were boiled with the protein loading buffer (without SH-group reductant) for 5 min and collected for western blot analysis.

### 2.12. GST Pull-Down

GST pull-down was performed following the manufacturer’s instructions for the Pierce GST Protein Interaction Pull-Down Kit (Thermo Fisher Scientific, Shanghai, China). Briefly, the plasmids pGEX-6p-1, pGEX-GST-PKM2, pGEX-GST-NS5B, and pGEX-GST-C were transformed into BL21 competent cells (Invitrogen, Shanghai, China) to obtain GST, GST-PKM2, GST-NS5B, and GST-C protein. The plasmids pcDNA3.1-PKM2-Flag, pcDNA3.1-NS5B-Flag, and pcDAN3.1-C-Flag were transfected into PK-15 cells to obtain the PKM2-Flag, NS5B-Flag, and C-Flag proteins. Then, BL21 cells were lysed by a pull-down lysis buffer and immobilized on equilibrated glutathione agarose resin at 4 °C for 2 h, followed by washing with a wash solution. Then, the resin was incubated with transfected PK-15 cells lysates at 4 °C for 12 h. After washing, the resin was eluted with glutathione elution buffer. The interaction between proteins was evaluated by western blot.

### 2.13. Luciferase Assay

Luciferase assay was performed based on the manufacturer’s instructions of the Dual-Luciferase Reporter Assay System (Promega, Beijing, China). Briefly, 293T cells were seeded in 12-well plates. Then, the plasmids encoding the dsRNA sensor RIG-I, renilla luciferase driven by thymidine kinase (TK) promoter and firefly luciferase driven by IFN-β promoter, and plasmid expressing viral NS5B wildtype or mutants were transfected into 293T cells. After 24 h, the culture medium was discarded, and cells were washed with PBS, after which cells were lysed using PLB lysate and placed in a shaker for 20 min. Next, Dual-Glo luciferase assay reagent was added to the plate for 2 s, and the luciferase reaction intensity was measured immediately. Then, Dual-Glo Stop & Glo Reagent was added to the plate for 2 s, and the luciferase reaction was detected immediately. The ratio of firefly to Renilla luminescence represents the amount of luciferase, normalized by the negative control.

### 2.14. Statistical Analysis

Data were analyzed using Excel and GraphPad Prism 6 software and expressed as mean ± standard deviation (SD). The significance analysis was performed by Student’s *t*-test. Differences were considered significant when *p* < 0.05.

## 3. Results

### 3.1. PKM2 Level Influences the Production of CSFV

**Figure 1:**
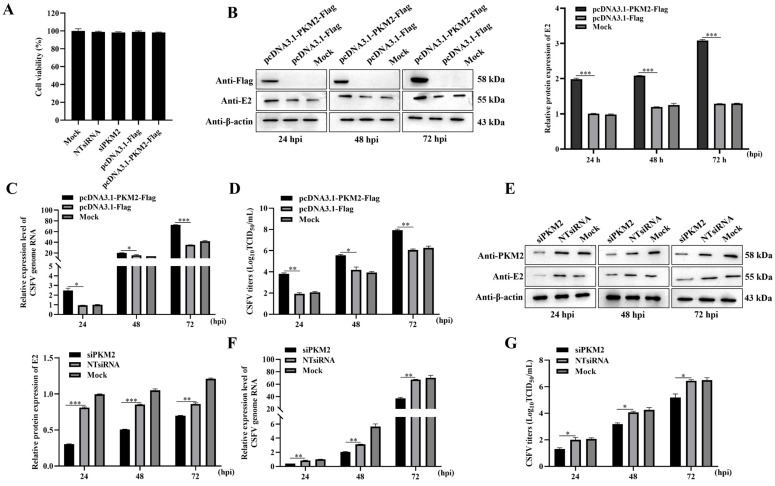
Overexpression and knockdown of PKM2 influences CSFV propagation. (**A**) The cell viability upon transfection of siRNA and plasmid was measured by the CCK-8 assay; (**B**–**D**) Overexpression of PKM2 in PK-15 cells by transfecting cells with pcDNA3.1-PKM2-Flag; (**E**–**G**) Knockdown of PKM2 in PK-15 cells using siRNA; (**B**,**E**) Western blot image to analyze the propagation of CSFV using an anti-E2 antibody. Cells were seeded in 6-well plates and transfected with siRNA and plasmid as indicated. After 24 h of transfection, cells were infected with CSFV (MOI = 1), and CSFV E2 protein expression was detected at indicated times using western blot assay. β-actin was an internal control. (**C**,**F**) RT-qPCR analyzes CSFV RNA levels. Cells were seeded in 12-well plates and transfected with siRNA and plasmid as indicated. After 24 h of transfection, cells were infected with CSFV (MOI = 1), and CSFV RNA level was detected at indicated times using RT-qPCR. β-actin mRNA level was used for normalization. (**D**,**G**) TCID_50_ assay detected CSFV viral titers in the supernatants of cells transfected with different plasmid or siRNA. Cells were transfected with siRNA and plasmid and then infected with CSFV (MOI = 1) after 24 h of transfection. The supernatants of cells were collected at indicated times for TCID_50_ assay. Results are illustrated as the mean ± SD (*n* = 3). *, *p* < 0.05; **, *p* < 0.01; ***, *p* < 0.001; ns, no significance (*p* > 0.05).

To elucidate the role of PKM2 in CSFV propagation, cellular PKM2 was either knocked down or overexpressed through transfection of synthesized siRNA targeting PKM2 (siPKM2) or a PKM2-expressing plasmid (pcDNA3.1-PKM2-Flag). CCK-8 assay was performed to assess the viability of transfected cells, and neither the transfection of siPKM2 or pcDNA3.1-PKM2-Flag reduced cell viability (Figure 1A). Transfected cells were then infected with CSFV (MOI = 1) at 24 h post transfection. At 24, 48, and 72 h post-infection, cells were collected and subjected to western blot analysis for measuring the viral protein levels. Our results showed that higher amounts of viral E2 protein were detected in cells overexpressing PKM2 than in controls at all the checked timepoints, indicating that PKM2 could enhance virus proliferation (Figure 1B). Consistent with the viral protein amount, CSFV genome copies quantified by RT-qPCR also showed increased virus abundances in PKM2 overexpressing groups at all three timepoints (Figure 1C). Next, progeny virus production was also measured by viral titers in the whole cell culture (Figure 1D), which clearly showed that overexpressed PKM2 facilitates CSFV propagation in PK-15 cells. Furthermore, we checked CSFV proliferation after knocking down PKM2 expression. With endogenous PKM2 protein depleted strongly by a synthetic siRNA, far less viral E2 protein (about 25 percent of the scrambled siRNA control) was detected (Figure 1E). Similarly, both the genome copies (Figure 1F) and virus titers (Figure 1G) are significantly reduced in siRNA-treated cells, demonstrating the indispensable roles of PKM2 in CSFV propagation. Taking together, these data revealed that PKM2 propels propagation of CSFV, and its depletion strongly impairs the virus proliferation.

### 3.2. CSFV Infection Upregulates the Expression of PKM2

**Figure 2:**
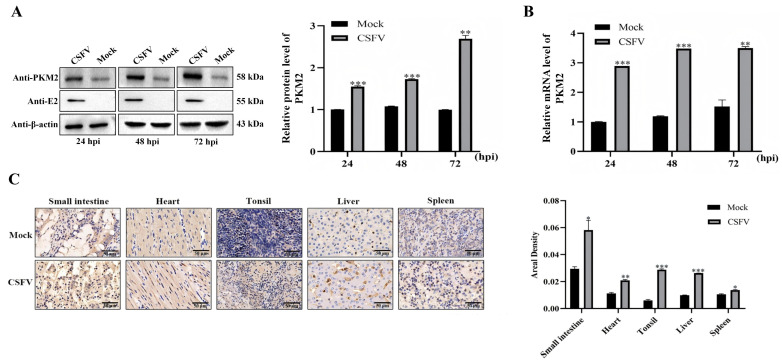
CSFV upregulates the expression of PKM2. (**A**) Western blot analyzed the protein level of PKM2. Cells were infected with CSFV (MOI = 1), and cellular PKM2 protein was detected at indicated times using western blot assay. β-actin was used for the internal control. (**B**) The mRNA level of PKM2 was detected by RT-qPCR, normalized by β-actin. (**C**) Piglets were infected with CSFV and then autopsied for IHC to detect the change in PKM2 level in tissues; the areal density of PKM2 was measured by IPP6.0 software with anti-PKM2 antibody. The PKM2 signal was brownish. Results are illustrated as the mean ± SD (*n* = 3). *, *p* < 0.05; **, *p* < 0.01; ***, *p* < 0.001; ns, no significance (*p* > 0.05).

As it is a common strategy that viruses upregulate host factors that promote virus proliferation for its propagation [16,17,18,19], we wondered whether CSFV adapts such mechanism and modulates the expression of host PKM2. PK-15 cells infected with CSFV (MOI = 1) were collected at 24, 48, and 72 h post-infection, and the expression of PKM2 was measured at both the mRNA level and the protein level. Cellular PKM2 protein was significantly upregulated after CSFV infection, with increased expression following the virus proliferation (Figure 2A), which is consistent with the change in PKM2 mRNA (Figure 2B). These results demonstrated that CSFV infection leads to upregulated PKM2 level in cultured cells. Next, the expression of PKM2 was checked in infected animals. Piglets were infected with moderate amounts of Shimen strain CSFV, and tissues such as kidneys and tonsils were sampled for IHC. In all checked tissues, heavier staining of PKM2 was observed in CSFV-infected pigs than in the control, and semi-quantification of the staining also confirmed significantly higher PKM2 levels in CSFV-infected tissues (Figure 2C), demonstrating that CSFV infection upregulates expression of host PKM2 in infected animals.

### 3.3. PKM2 Binds to CSFV NS5B Independent of Viral C Protein

**Figure 3:**
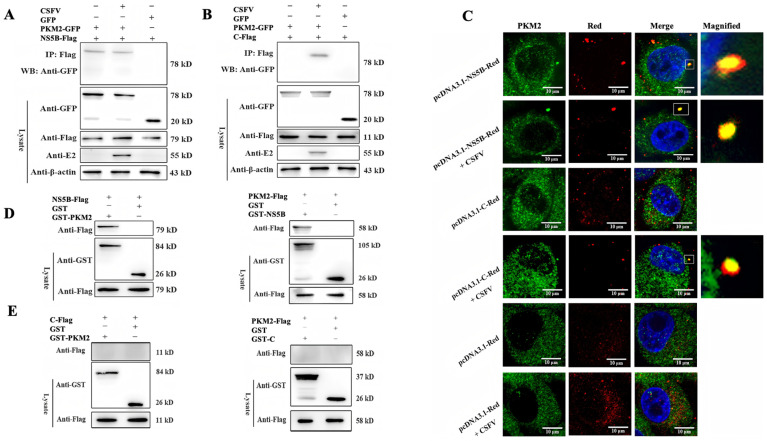
PKM2 directly binds to CSFV NS5B. (**A**,**B**) Co-immunoprecipitation (co-IP) assay. PK-15 cells were co-transfected with indicated plasmids and (or) infected with CSFV (MOI = 1) after 24 h. At 48 h after transfection, cells were lysed and immunoprecipitated, and western blot analysis was conducted using anti-GFP, anti-Flag, anti-E2, and anti-β-actin antibodies. (**C**) Detection of colocalization between PKM2 and NS5B by confocal microscopy. PKM2 was detected by an anti-PKM2 (green) antibody, while NS5B is marked by fused RFP (red). Scale bars, 10 μm. (**D**,**E**) Detection of the interaction by GST pull-down assay. GST, GST-PKM2, GST-C, or GST-NS5B fusion proteins expressed in *E. coli* BL21 (DE3) were purified with glutathione agarose and incubated with the lysate of relative transfected cells. Western blot analyzed the relationship between each protein using anti-GST and anti-Flag antibodies.

The PKM gene is found in vertebrates and is highly conservative in different species, and the PKM2 from humans has been shown to interact with and regulate the function of several viral RdRps [11,20] To check whether pig PKM2 also binds to NS5B, which is the RdRp of CSFV, Co-IP experiments were performed. PKM2-GFP fusion protein was co-precipitated with NS5B protein, confirming the interaction between the two proteins. Additionally, the interaction could be observed in both the presence and absence of CSFV infection, indicating that other viral proteins might not be required to mediate the interaction between the two proteins (Figure 3A). PKM2 and CSFV C protein have previously been found in the same complex in CSFV-infected cells by IP-MS, and we examined the potential interaction between PKM2 and C protein. The C protein was co-precipitated with NS5B only in CSFV infection, suggesting an indirect interaction between them mediated by other viral or cellular proteins (Figure 3B) [21]. Next, we check the cellular co-localization of the proteins by confocal microscopy. Consistent with the Co-IP results, while NS5B showed good colocalization with PKM2 in CSFV-infected or uninfected cells, the C protein colocalized with PKM2 only in the presence of CSFV infection, indicating an indirect interaction between the two proteins (Figure 3C). Based on these observations, GST pull-down assays were performed by expression and purification of the indicated proteins in PK-15 cells or BL-21 *E. coli*. While NS5B showed a direct interaction with PKM2 (Figure 3D), no interaction was detected between the purified C and PKM2 protein (Figure 3E). All together, these data demonstrated that PKM2 directly interacts with NS5B but not the C protein of CSFV.

### 3.4. PKM2 Increases the Replication of the CSFV Genome

**Figure 4:**
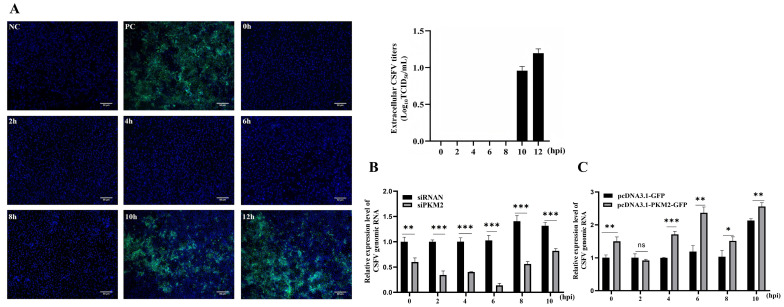
PKM2 increases CSFV genome replication. (**A**) determination of temporal parameters for the progeny virus release. Cells were infected with CSFV (MOI = 1), and supernatants of infected cells were collected at indicated times for 12 h to detect the extracellular CSFV titers. NC, non-infection control; PC, CSFV inoculated positive control; scale bars, 50 μm. (**B**,**C**) The change in PKM2 level influences CSFV genome replication before the release of progeny virions. Cells were seeded in 12-well plates and transfected with siRNA or pcDNA3.1-PKM2-Flag and then infected with CSFV. At 2, 4, 6, 8, and 10 h post infection, the genomic RNA amount was detected by RT-qPCR, normalized by β-actin. Results are illustrated as the mean ± SD (*n* = 3). *, *p* < 0.05; **, *p* < 0.01; ***, *p* < 0.001; ns, no significance (*p* > 0.05).

Since the major function of NS5B is viral RNA synthesis and replication, we were wondering about the potential effects of PKM2-NS5B interaction on viral genome replication. To investigate the impact of PKM2 on viral genome replication while avoiding progeny virus reinfection-mediated amplification of proliferation differences during prolonged cultivation, we specifically examined the temporal parameters for the first progeny virus release after CSFV infection. Following viral inoculation at MOI 1, cell culture supernatants were collected every 2 h for a duration of 12 h to check whether the progeny virus was secreted. The supernatant was then inoculated into a PK-15 cell, and the secreted virus was confirmed by detection of the viral E2 protein 24 h after inoculation, which revealed that the secretion of progeny virions begins at 10 hours post-infection, establishing the first reproduction cycle duration as approximately 10 hours (Figure 4A). Subsequent evaluation of viral RNA replication showed that PKM2 knockdown resulted in reduced CSFV RNA genome levels across all measured time points compared to controls (Figure 4B). Conversely, PKM2 overexpression markedly enhanced viral RNA levels, with experimental groups showing statistically significant higher RNA levels than control groups (Figure 4C). These findings demonstrate that PKM2 facilitates CSFV genome replication during the infection cycle.

### 3.5. Evidence for a PKM2 Effect on NS5B Activity

**Figure 5:**
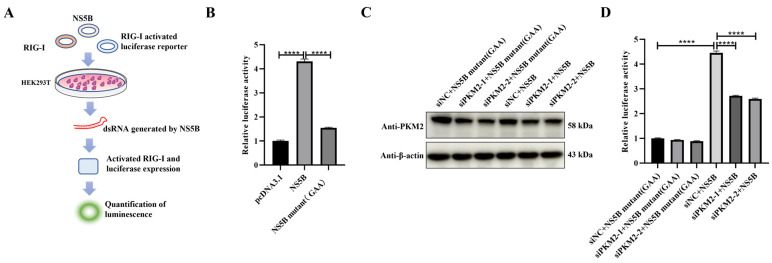
PKM2 enhances NS5B RdRp activity. (**A**) Schematic illustration of the cell-based indirect detection method for CSFV NS5B RdRp activity. Plasmids were triple-expressed into cells; the RdRp activity of NS5B generates dsRNA, which is recognized by RIG-I protein, thus activating the RIG-I signaling pathway and subsequently inducing firefly luciferase expression driven by the IFN-β promoter in downstream signaling. (**B**) Comparison of firefly to Renilla luciferase ratios across different groups after transient plasmid transfection into HEK293T cells to assess NS5B RdRp activity. NS5B mutant (GAA) represents an enzymatically inactive mutant of CSFV NS5B. (**C**) Cellular PKM2 detected by western blot after siRNA-mediated knockdown. Less cellular PKM2 was blotted in groups treated with siPKM2-1 or siPKM2-2 than in the nontargeting control siRNA (siNC)-treated groups. (**D**) Dual-luciferase assay evaluating the impact of PKM2 knockdown by siRNA on NS5B RdRp activity. Two effective siRNA sequences demonstrating potent PKM2 interference efficiency were selected for this investigation. Results are presented as the mean ± SD (*n* = 3). ****, *p* < 0.0001.

So far, we have shown that PKM2 binds to CSFV NS5B and increases the replication of the CSFV genome. As NS5B is the virus RNA polymerase responsible for viral genomic RNA synthesis, we were wondering whether PKM2 promoted CSFV proliferation by modulating the efficiency of NS5B via the direct interaction between the two proteins. Therefore, a dual luciferase assay was performed to detect viral RdRp activity [22,23]. In this assay, dsRNA produced by NS5B activates the co-transfected RIG-1 in HEK293T cells and therefore activates a firefly luciferase reporter controlled by the activity of RIG-1. The luciferase reporter was measured with a luminescence reader and normalized by Renilla luciferase under a CMV promoter, indirectly representing the activity of NS5B (Figure 5A). The activity of NS5B as well as a catalytic inactive mutant (GDD to GAA) was measured by this method, and the wildtype NS5B showed significantly higher luminescence than the GAA mutant, supporting successful measurement of NS5B activity (Figure 5B). Next, endogenous cellular PKM2 was knocked down by siRNA (Figure 5C), the activity of NS5B as well as that of the catalytic inactive mutant were evaluated, and a significant reduction in luciferase activity (about 50%) was observed when PKM2 was knocked down, indicating that PKM2 facilitates the NS5B-mediated dsRNA formation (Figure 5D). These data demonstrated that PKM2 is required for the full activity of NS5B, suggesting that PKM2 enhances the activity of NS5B.

## 4. Discussion

In recent years, research on the interplay between cellular metabolism and viral pathogenesis has gained much attention. Glucose metabolism has been demonstrated to play pivotal roles in regulating multiple stages of viral life cycles [24]. This study investigates the potential function of glucose metabolic enzyme PKM2 in CSFV proliferation. Our study reveals that CSFV infection significantly induces transcriptional upregulation of PKM2 in host cells and PKM2 enhances viral genomic replication efficiency through direct protein–protein interaction with the viral NS5B protein. Moreover, the gene reporter assay indicates that PKM2 markedly potentiates the catalytic activity of the NS5B protein, offering new insights into the mechanism of PKM2-facilitated CSFV propagation. These findings establish PKM2 as an important factor bridging host glycolytic flux and CSFV replication, offering new insights into virus infection and glucose metabolism. 

As an essential glycolytic enzyme, pyruvate kinase is necessary for the production of ATP. While the M1 isoform of the M-type pyruvate kinase is stably expressed for basic-level glycolysis in many tissues, the M2 isoform acts as a metabolic sensor to balance energy production with the production of other biosynthetic precursors [25,26,27]. Therefore, several studies have shown that PKM2 is involved in virus proliferation in both DNA and RNA viruses [28,29,30]. For CSFV, a previous study showed that PKM2 promoted CSFV propagation by promoting expression of NS4A and NS5A and also by regulating the mitophagy pathway [14]. The catalytic activity of PKM2 was shown to be involved, and the pyruvate products were necessary for such effects of PKM2 on CSFV proliferation, revealing an indirect action of PKM2 on the propagation of CSFV. Here, our research showed a direct effect of PKM2 on enhancing the activity of the NS5B protein, therefore propelling virus genome synthesis, our finding further expanding the direct function of this key metabolic enzyme on CSFV proliferation.

In a previous study, PKM2 was shown to be co-immunoprecipitated with CSFV NS4A and NS5A [14], and here we found that PKM2 also interacts with and enhances the activity of the viral NS5B protein, facilitating CSFV RNA synthesis. The viral proteins NS4A, NS5A, and NS5B could co-exist in the same protein complex for virus replication in HCV [31], and our data showed a direct interaction between PKM2 and CSFV NS5B protein via a GST pull-down assay in bacteria. Whether PKM2 interacts with NS4A and NS5A directly or indirectly by bridging NS5B is still unclear, which would be interesting to check for understanding the potential connection between these two independent investigations, thus offering a full view of the function and mechanism of PKM2 in CSFV propagation.

PKM2 was shown to interact with the NS5B protein of HCV, which belongs to the same family as CSFV. Binding of PKM2 to HCV NS5B changed the conformation of HCV NS5B, enhancing its polymerase activity, which is similar to our finding here. Infection of another member of the Flaviviridae, Dengue virus, leads to phosphorylation of PKM2, which enhances glycolysis for virus propagation [32,33]. In influenza A virus, PKM2 promotes assembly of the RNA polymerase complex composed of PA, PB1, and PB2 [11,34]. These studies suggest that modulation of the RdRp activity might be common for PKM2 in regulating virus infection. Further study on the structural basis for PKM2 and RdRp interaction is required to test this idea.

In conclusion, we revealed that CSFV infection upregulates expression of PKM2 in cell culture as well as in infected animals, and host PKM2 increased CSFV replication by enhancing the RdRp activity of NS5B. Our findings showed a new mechanism of host factors regulating CSFV infection and provided new information on investigating host pyruvate kinase and virus interactions.

## Data Availability

All relevant data are within the paper and supplementary data.

## Supplementary Materials

The following supporting information can be downloaded at: https://www.mdpi.com/article/10.3390/v17050648/s1, Table S1. List of primers used in this study.

## Abbreviations

The following abbreviations are used in this manuscript:

CSFV	classical swine fever virus
PKM2	pyruvate kinase M2
NS5B	non-structural protein 5B
RdRp	RNA-dependent RNA polymerase

## References

[1] ICTV https://ictv.global/taxonomy.

[2] Ji W., Guo Z., Ding N.Z., He C.Q. (2015). Studying classical swine fever virus: Making the best of a bad virus. Virus Res..

[3] Li S., Wang J., Yang Q., Naveed A.M., Yu S., Qiu H.J. (2017). Complex Virus-Host Interactions Involved in the Regulation of Classical Swine Fever Virus Replication: A Minireview. Viruses.

[4] Xiao M., Gao J., Wang W., Wang Y., Chen J., Chen J., Li B. (2004). Specific interaction between the classical swine fever virus NS5B protein and the viral genome. Eur. J. Biochem..

[5] Tautz N., Kaiser A., Thiel H.J. (2000). NS3 serine protease of bovine viral diarrhea virus: Characterization of active site residues, NS4A cofactor domain, and protease-cofactor interactions. Virology.

[6] Xiao M., Wang Y., Chen J., Li B. (2003). Characterization of RNA-dependent RNA polymerase activity of CSFV NS5B proteins expressed in Escherichia coli. Virus Genes.

[7] Schormann N., Hayden K.L., Lee P., Banerjee S., Chattopadhyay D. (2019). An overview of structure, function, and regulation of pyruvate kinases. Protein Sci..

[8] Israelsen W.J., Vander H.M. (2015). Pyruvate kinase: Function, regulation and role in cancer. Semin. Cell Dev. Biol..

[9] Alquraishi M., Puckett D.L., Alani D.S., Humidat A.S., Frankel V.D., Donohoe D.R., Whelan J., Bettaieb A. (2019). Pyruvate kinase M2: A simple molecule with complex functions. Free Radic. Biol. Med..

[10] Wu X., Zhou Y., Zhang K., Liu Q., Guo D. (2008). Isoform-specific interaction of pyruvate kinase with hepatitis C virus NS5B. FEBS Lett..

[11] Miyake Y., Ishii K., Honda A. (2017). Influenza Virus Infection Induces Host Pyruvate Kinase M Which Interacts with Viral RNA-Dependent RNA Polymerase. Front. Microbiol..

[12] Chuang C., Prasanth K.R., Nagy P.D. (2017). The Glycolytic Pyruvate Kinase Is Recruited Directly into the Viral Replicase Complex to Generate ATP for RNA Synthesis. Cell Host Microbe.

[13] Gou H., Zhao M., Yuan J., Xu H., Ding H., Chen J. (2017). Metabolic Profiles in Cell Lines Infected with Classical Swine Fever Virus. Front. Microbiol..

[14] Liu X., Yan Q., Liu X., Wei W., Zou L., Zhao F., Zeng S., Yi L., Ding H., Zhao M. (2024). PKM2 induces mitophagy through the AMPK-mTOR pathway promoting CSFV proliferation. J. Virol..

[15] Liu S. (2021). Effects of Host PKM Protein on CSFVProliferation and Interaction with NS5B. Master’ Thesis.

[16] Gong X., Li X., You X., Hu A., Liu M., Yao H., He J., Zhang X., Ning P. (2020). AIF1 was identified as an up-regulated gene contributing to CSFV Shimen infection in porcine alveolar macrophage 3D4/21 cells. PeerJ.

[17] Jung G.S., Jeon J.H., Choi Y.K., Jang S.Y., Park S.Y., Kim S.W., Byun J.K., Kim M.K., Lee S., Shin E.C. (2016). Pyruvate dehydrogenase kinase regulates hepatitis C virus replication. Sci. Rep..

[18] Mouree K.R., Kishimoto N., Iga N., Kirihara C., Yamamoto K., Takamune N., Misumi S. (2018). Virion-Packaged Pyruvate Kinase Muscle Type 2 Affects Reverse Transcription Efficiency of Human Immunodeficiency Virus Type 1 by Blocking Virion Recruitment of tRNA(Lys3). Biol. Pharm. Bull..

[19] Kirchhoff F., Schindler M., Bailer N., Renkema G.H., Saksela K., Knoop V., Müller-Trutwin M.C., Santiago M.L., Bibollet-Ruche F., Dittmar M.T. (2004). Nef proteins from simian immunodeficiency virus-infected chimpanzees interact with p21-activated kinase 2 and modulate cell surface expression of various human receptors. J. Virol..

[20] Zheng Z., Zhou J., Song Y. (2024). Safety of RNA-Dependent RNA Polymerase Inhibitors, Molnupiravir and VV116, for Oral Treatment of COVID-19: A Meta-Analysis. Iran. J. Med. Sci..

[21] Li W., Zhang Y., Kao C.C. (2014). The classic swine fever virus (CSFV) core protein can enhance de novo-initiated RNA synthesis by the CSFV polymerase NS5B. Virus Genes.

[22] She Y., Liao Q., Chen X., Ye L., Wu Z. (2008). Hepatitis C virus (HCV) NS2 protein up-regulates HCV IRES-dependent translation and down-regulates NS5B RdRp activity. Arch. Virol..

[23] She Y., Han T., Ye L., Wu Z. (2009). Hepatitis C virus NS2/3 protease regulates HCV IRES-dependent translation and NS5B RdRp activity. Arch. Virol..

[24] Li H., Lin C., Qi W., Sun Z., Xie Z., Jia W., Ning Z. (2023). Senecavirus A-induced glycolysis facilitates virus replication by promoting lactate production that attenuates the interaction between MAVS and RIG-I. PLoS Pathog..

[25] Grant M.M. (2021). Pyruvate Kinase, Inflammation and Period.d.dontal Disease. Pathogens.

[26] Anastasiou D., Yu Y., Israelsen W.J., Jiang J., Boxer M.B., Hong B.S., Tempel W., Dimov S., Shen M., Jha A. (2012). Pyruvate kinase M2 activators promote tetramer formation and suppress tumorigenesis. Nat. Chem. Biol..

[27] Wu B., Liang Z., Lan H., Teng X., Wang C. (2024). The role of PKM2 in cancer progression and its structural and biological basis. J. Physiol. Biochem..

[28] Lee Y.B., Min J.K., Kim J.G., Cap K.C., Islam R., Hossain A.J., Dogsom O., Hamza A., Mahmud S., Choi D.R. (2022). Multiple functions of pyruvate kinase M2 in various cell types. J. Cell Physiol..

[29] Ren X., Song H., Wang Y., Wang Y., Zhang Q., Yue X., Wu Z., Li C., Gao L., Ma C. (2024). TIPE1 limits virus replication by disrupting PKM2/ HIF-1α/ glycolysis feedback loop. Free Radic. Biol. Med..

[30] Lo A.K., Dawson C.W., Young L.S., Ko C.W., Hau P.K., Lo K.W. (2015). Activation of the FGFR1 signalling pathway by the Epstein-Barr virus-encoded LMP1 promotes aerobic glycolysis and transformation of human nasopharyngeal epithelial cells. J. Pathol..

[31] Lin C., Wu J.W., Hsiao K., Su M.S. (1997). The hepatitis C virus NS4A protein: Interactions with the NS4B and NS5A proteins. J. Virol..

[32] Wongtrakul J., Thongtan T., Pannengpetch S., Wikan N., Kantamala D., Kumrapich B., Suwan W., Smith D.R. (2020). Phosphoproteomic analysis of dengue virus infected U937 cells and identification of pyruvate kinase M2 as a differentially phosphorylated phosphoprotein. Sci. Rep..

[33] Fontaine K.A., Sanchez E.L., Camarda R., Lagunoff M. (2015). Dengue virus induces and requires glycolysis for optimal replication. J. Virol..

[34] Ren L., Zhang W., Zhang J., Zhang J., Zhang H., Zhu Y., Meng X., Yi Z., Wang R. (2021). Influenza A Virus (H1N1) Infection Induces Glycolysis to Facilitate Viral Replication. Virol. Sin..

