# Supplementary material for: PKM2 Facilitates Classical Swine Fever Virus Replication by Enhancing NS5B Polymerase Function
Source: Viruses. 2025 Apr 29;17(5):648. doi: 10.3390/v17050648 (PMC12115705; doi:10.3390/v17050648)
Supplement: Supplementary file 1 [file viruses-17-00648-s001.zip › viruses-3573225-supplementary.pdf]

Table S1. List of primers used in this study.

Primer name	Sequence (5′-3′)	Purpose
RT-β-actin-sense	CAAGGACCTCTACGCCAACAC	Quantitative Real-time PCR
RT-β-actin-antisense	TGGAGGCGCGATGATCTT	
RT-CSFV-sense	GATCCTCATACTGCCCACTTAC	
RT-CSFV-antisense	GTATACCCCTTCACCAGCTTG	
RT-PKM2-sense	CCTGGGAGAGAAAGGAAAGAAC	
RT-PKM2-antisense	CCACGAGCCACCATGATAC	
pcDNA3.1-PKM2-sense	CGCGGATCCGCCACCATGCCGAAGCCCCACAG	PCR amplification primers for the PKM2 gene
pcDNA3.1-PKM2-antisense	CCGGAATTCCACGGCACAGGCACTACGC	
6P-1-PKM2-sense	CGCGGATCCATGCCGAAGCCCCACAG	
6P-1-PKM2-antisense	CCGCTCGAGTCACGGCACAGGCACTACGC	
siPKM2-sense1	GGAAUGAACGUGGCUCGUUTT	Small interfering RNAs (siRNAs) for PKM2
siPKM2-antisense1	AACGAGCCACGUUCAUUCCTT	
siPKM2-sense2	CCAUCUACCAUUUGCAAUUTT	
siPKM2-antisense2	AAUUGCAAAUGGUAGAUGGTT	
siPKM2-sense3	CCCGAGGCUUCUUCAAGAATT	
siPKM2-antisense3	UUCUUGAAGAAGCCUCGGGTT	

Note: The underlined sequence indicates the restriction enzyme site.
